# ClearScope: A Fully Integrated Light-Sheet Theta Microscope for Sub-Micron-Resolution Imaging Without Lateral Size Constraints

**DOI:** 10.3390/jimaging12030118

**Published:** 2026-03-10

**Authors:** Matthew G. Fay, Peter J. Lang, David S. Denu, Nathan J. O’Connor, Benjamin Haydock, Jeffrey Blaisdell, Nicolas Roussel, Alissa Wilson, Sage R. Aronson, Veronica Pessino, Paul J. Angstman, Cheng Gong, Tanvi Butola, Orrin Devinsky, Jayeeta Basu, Raju Tomer, Jacob R. Glaser

**Affiliations:** 1 MBF Bioscience, Williston, VT 05495, USA; mfay@mbfbioscience.com (M.G.F.); peter@mbfbioscience.com (P.J.L.); dave@tek5systems.com (D.S.D.); nathan@arnastech.com (N.J.O.); ben@mbfbioscience.com (B.H.); jblaisdell@mbfbioscience.com (J.B.); nicolasr@mbfbioscience.com (N.R.); alissa@mbfbioscience.com (A.W.); sage@kymo.io (S.R.A.); vpessino@mbfbioscience.com (V.P.); paul@mbfbioscience.com (P.J.A.); 2 Department of Biomedical Engineering, Columbia University, New York, NY 10027, USA; cheng.gong@columbia.edu (C.G.); raju.tomer@columbia.edu (R.T.); 3 Department of Biological Sciences, Columbia University, New York, NY 10027, USA; 4 Neuroscience Institute, New York University Langone Health, New York, NY 10016, USA; tanvi.butola@nyulangone.org (T.B.); jayeeta.basu@nyulangone.org (J.B.); 5 Department of Neuroscience and Physiology, New York University Grossman School of Medicine, New York, NY 10016, USA; orrin.devinsky@nyulangone.org; 6 Comprehensive Epilepsy Center, Department of Neurology, New York University Grossman School of Medicine, New York, NY 10016, USA; 7 Department of Psychiatry, New York University Grossman School of Medicine, New York, NY 10016, USA; 8 Department of Neurosurgery, New York University Grossman School of Medicine, New York, NY 10016, USA; 9 Center for Neural Science, New York University, New York, NY 10003, USA

**Keywords:** light-sheet microscopy, light-sheet theta microscopy, connectomics, neuroscience

## Abstract

Three-dimensional (3D) ex vivo imaging of cleared tissue from intact brains from animal models, human brain surgical specimens, and large postmortem human and non-human primate brain specimens is essential for understanding physiological neural connectivity and pathological alterations underlying neurological and neuropsychiatric disorders. Contemporary light-sheet microscopy enables rapid, high-resolution imaging of large, cleared samples but is limited by the orthogonal arrangement of illumination and detection optics, which constrains specimen size. Light-sheet theta microscopy (LSTM) overcomes this limitation by employing two oblique illumination paths while maintaining a perpendicular detection geometry. Here, we report the development of a next-generation, fully integrated and user-friendly LSTM system that enables uniform subcellular-resolution imaging (with subcellular resolution determined by the lateral performance of the system) throughout large specimens without constraining lateral (XY) dimensions. The system provides a seamless workflow encompassing image acquisition, data storage, pre- and post-processing, enhancement and quantitative analysis. Performance is demonstrated by high-resolution 3D imaging of intact mouse brains and human brain samples, including complete downstream analyses such as digital neuron tracing, vascular reconstruction and design-based stereological analysis. This enhanced and accessible LSTM implementation enables rapid quantitative mapping of molecular and cellular features in very large biological specimens.

## 1. Introduction

Three-dimensional (3D) ex vivo whole-brain imaging of transgenic and non-transgenic animal models and human brain residual surgical tissue, as well as large postmortem human and non-human primate brain specimens, holds substantial potential for gaining novel insights into both physiological neural network connectivity and pathological alterations associated with neuropsychiatric and neurological disorders [1,2,3].

Among available imaging modalities, light-sheet microscopy (LSM) has emerged as the most effective approach for whole-brain imaging compared to confocal and multiphoton microscopy, as it combines high 3D resolving power with rapid data acquisition and reduced phototoxicity [4]. LSM achieves optimal performance when combined with tissue-clearing techniques—including CLARITY [5,6,7], CUBIC [8], iDISCO [9], uDISCO [10], SeeDB [11], Scale [12] and Binaree Tissue Clearing [13]—which enable optical access to intact tissue while preserving molecular and structural architecture, particularly long-range neuronal connections.

More than 30 LSM implementations have been described in the literature [4], yet virtually all face critical constraints when imaging large specimens. Specifically, in conventional orthogonal LSM designs, the illumination attenuates when specimen lateral dimensions exceed 10–15 mm, approximately the size of a mouse brain, making it impossible to image intact large rodent brains, human brain tissue sections, or other clinically relevant samples in their entirety (discussed in [14]). Alternative selective plane illumination microscopy (SPIM) configurations avoid this collision problem by orienting the detection objective at an oblique angle (e.g., 45°) to the specimen surface or by using lower numerical aperture objectives with increased clearance. However, angled detection severely limits maximum imaging depth, as the working distance is consumed more rapidly, and it introduces asymmetric optical aberrations that degrade image quality, particularly at greater depths where refractive index mismatches become problematic (also addressed in [14]). Lower-magnification approaches sacrifice the resolution needed to identify individual neurons and subcellular structures. These constraints force researchers to sacrifice either spatial extent (by sectioning specimens), imaging depth (by using angled detection) or resolution (by using lower-performance optics), all of which compromise the core advantages of 3D high-resolution imaging.

Recently, light-sheet theta microscopy (LSTM) was introduced as a novel LSM architecture [14]. LSTM employs two illumination paths oriented obliquely to a detection path that is arranged perpendicular to the specimen. This geometry enables several capabilities that are not simultaneously available in existing LSM systems, including: (i) imaging of thick specimens with effectively unlimited lateral (XY) extent and a depth (Z) range constrained only by the working distance of the detection objective; (ii) simultaneous dual-angle illumination to ensure excitation of regions shadowed by opaque structures; (iii) independent selection of illumination and detection objectives with different magnifications and numerical apertures for optimized imaging performance; and (iv) compatibility with objectives of different refractive indices and from multiple manufacturers, allowing flexibility across tissue-clearing protocols and future objective innovations.

Proof of concept for LSTM was established in [14], demonstrating that the technology represents a significant advance beyond the state of the art. Building on this foundation, the present project aimed to further develop LSTM by designing and testing: (i) an optimized microscope architecture; (ii) a novel specimen and immersion-medium chamber; (iii) a detection objective changer; (iv) advanced image acquisition software with adaptive refractive index mismatch correction; and (v) software for stitching LSTM image stacks into composite 3D datasets without the need for downsampling.

The ultimate goal of this project was to develop a novel, fully integrated light-sheet theta microscope (ClearScope^®^), designed such that the configuration of the illumination and detection objectives enables subcellular-resolution imaging (with subcellular resolution determined by the lateral performance of the system) of specimens with unconstrained lateral (XY) dimensions and a depth (Z) range limited only by the working distance of the detection objective used. In parallel, the project sought to deliver a seamless end-to-end workflow integrating image acquisition, data storage, post-processing, enhancement and quantitative analysis, thereby enabling advanced light-sheet microscopy without requiring specialized expertise in microscope hardware assembly or software programming.

## 2. Materials and Methods

**Figure 1:**
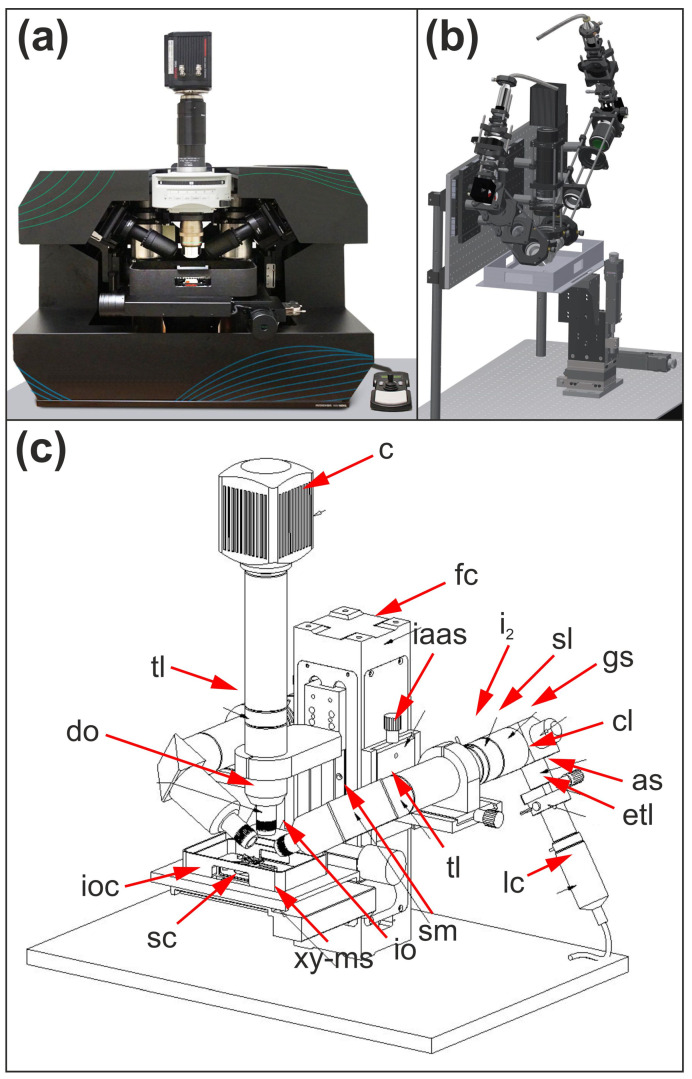
Overview of the ClearScope microscope and its predecessor. (**a**) Photograph of the production version of ClearScope, showing the compact, single-box design that integrates the illumination arms, detection arm, focus column, motorized stage and specimen holding components. (**b**) 3D computer-aided design (CAD) sketch of the original LSTM system [14], illustrating the fundamental optical and mechanical layout on which the ClearScope design is based. (**c**) 3D CAD drawing of ClearScope, highlighting the refined optical paths and modular hardware architecture, including the dual illumination arms, detection optics, focus column and specimen chamber within the immersion oil environment. Abbreviations: as, adjustable slit; c, camera; cl, cylindrical lens; do, detection objective; etl, electrically tunable lens; fc, focus column; gs, galvo scanner; i_2_, second iris; iaas, illumination light arm alignment slide; io, illumination objective; ioc, immersion oil chamber; lc, laser collimator; sc, specimen chamber; sl, f-theta scan lens; sm, steering mirror; tl, tube lens; xy-ms, XY motorized stage. Panel (**b**) is reproduced without modification from [14], published under the CC BY 4.0 license URL: https://creativecommons.org/licenses/by/4.0/ (accessed on 17 January 2025).

The ClearScope system (MBF Bioscience, Williston VT, USA) was developed as a turn-key product in a compact single-box design, intended to simplify shipment, installation and operation in standard laboratory environments. Where possible, the system is self-aligning to enable stable, unattended (“lights-out”) operation. The hardware consists of two illumination arms, a detection arm, a focus column, a system base, a motorized stage, specimen holding assemblies and a system enclosure (Figure 1).

This modular architecture facilitates manufacturability, serviceability and scalability. Several sub-assemblies were outsourced to contractors with specialized expertise; for example, illumination arm alignment was performed by an optical assembly company. Shipping brackets were designed to immobilize and brace the system during transport.

**Figure 2:**
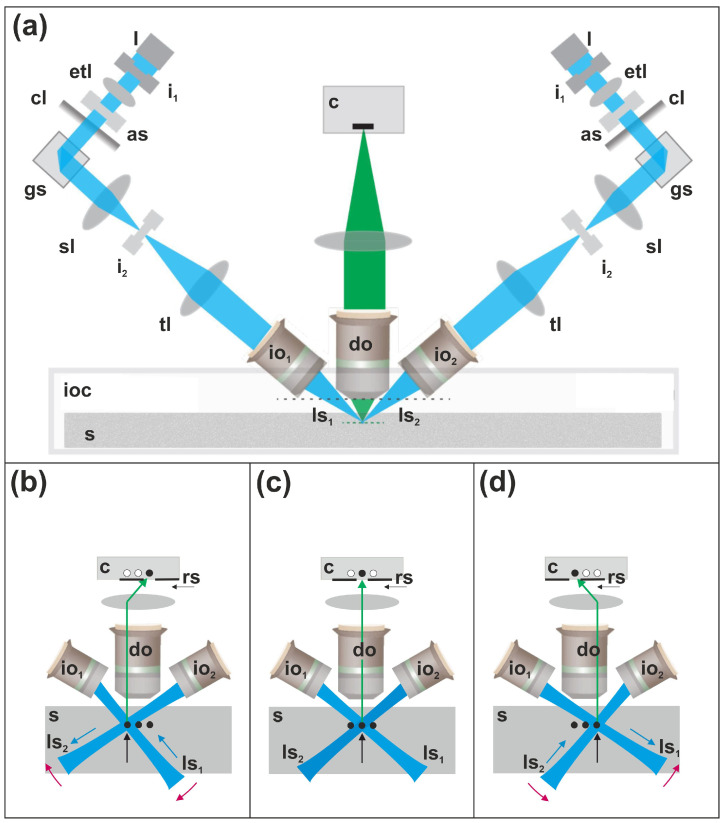
Principle of image acquisition with the ClearScope microscope. (**a**) Schematic overview of the optical layout of the ClearScope illumination and detection paths, showing laser delivery, beam shaping, dual light-sheet generation and detection through the rolling-shutter camera. (**b**) Imaging of a line on the left side of a single field of view (vertical black arrow in (**b**)), achieved by rotating both light sheets clockwise (red arrows in (**b**)) using the galvo scanners and translating the first light sheet into the first illumination objective (blue arrow marked by ls_1_ in (**b**)) while translating the second light sheet out of the second illumination objective (blue arrow marked by ls_2_ in (**b**)). (**c**) Imaging of a line in the center of the field of view (vertical black arrow in (**c**)), with both light sheets symmetrically aligned and positioned for on-axis illumination relative to the detection objective. (**d**) Imaging of a line on the right side of the field of view (vertical black arrow in (**d**)), achieved by rotating both light sheets counterclockwise (red arrows in (**d**)) and translating the first light sheet out of the first illumination objective (blue arrow marked by ls_1_ in (**d**)) while translating the second light sheet into the second illumination objective (blue arrow marked by ls_2_ in (**d**)). The left, right and center lines are represented by the three dots in the sample and the corresponding camera plane. Abbreviations: as, adjustable slit; c, camera; cl, cylindrical lens; do, detection objective; etl, electrically tunable lens; gs, galvo scanner; i_1_, first iris; i_2_, second iris; io_1_, first illumination objective; io_2_, second illumination objective; ioc, immersion oil chamber; l, laser; ls_1_, first light sheet; ls_2_, second light sheet; rs, rolling shutter; s, specimen; sl, f-theta scan lens; tl, tube lens. Panel (**a**) was modified from [14], published under the CC BY 4.0 license URL: https://creativecommons.org/licenses/by/4.0/ (accessed on 17 January 2025).

In the illumination path, laser light passes through a laser collimator, first iris, electrically tunable lens, adjustable slit, cylindrical lens, galvo scanner, f-theta scan lens, second iris, tube lens and illumination objective. Imaging is performed using two light sheets that are dynamically positioned and angled (Figure 2). Note: (1) The optical power at the specimen is between 1–15 mW, depending on hardware adjustments specific to each experiment. (2) Photobleaching was evaluated empirically in [14] by tracking how the average fluorescence signal changed over the course of each imaging acquisition. For every dataset, the authors plotted the mean signal intensity of sequentially acquired tiles in the order they were imaged (Figure 3f in [14]). If photobleaching were occurring, later tiles would show a systematic decrease in signal. As reported in [14], no such downward trend was observed, indicating that LSTM produced minimal photobleaching under their imaging conditions.

During operation, the specimen chamber is filled with media specific to the selected tissue-clearing protocol (details are provided below). The specimen chamber is then immersed in a refractive index liquid reservoir (RI = 1.4587); this media was selected to be the median of the typical range of cleared tissue refractive indices (RI = 1.33–1.56). Light-sheet scanning is controlled by galvanometric (galvo) scanners, while axial translation of the light sheets into and out of the illumination objectives is achieved using electrically tunable lenses (ETLs).

During microscope alignment, the ETLs are automatically stepped across the field of view (FOV) for each illumination light sheet, and the resulting focus profiles are used to adjust the ETL settings as a function of position within the FOV, thereby maintaining optimal focus across the entire imaging area. Synchronization of these components enables sequential imaging of multiple lines within a focal plane using a rolling shutter, as illustrated in Figure 2b–d.

The detection optical path supports a range of long-working-distance objectives, including an MBF Bioscience-modified UPLFLN4XPH objective (4×/0.13 NA; modified working distance 12 mm; Evident, Tokyo, Japan), an XLPLN10XSVMP objective (10×/0.6 NA; working distance 8 mm; Evident) and a CFI90 20XC Glyc objective (20×/1.0 NA; working distance 8.2 mm; Nikon, Tokyo, Japan).

Note: Modification of the UPLFLN4XPH objective (Evident) involved the construction of an immersion housing for the air objective, incorporating a fused silica plano-convex lens with the convex surface facing the objective and the planar surface interfacing with the immersion oil. This design follows the same optical principle illustrated in Figure 1d of [15].

**Figure 3:**
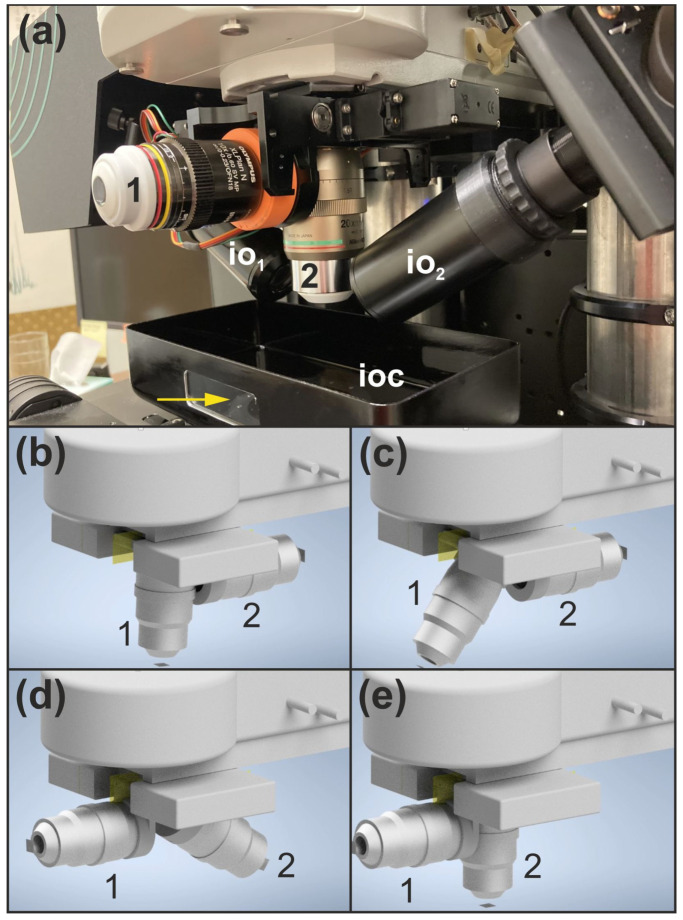
Detection objective changer (DOC) of the ClearScope microscope. (**a**) Detailed view of the DOC assembly, showing two mounted detection objectives, the relative positions of the illumination objectives and the immersion oil chamber; the yellow arrow indicates a window in the immersion oil chamber through which the light sheets can be observed. (**b**) DOC in the load position, with the system prepared for detection objective exchange. (**c**) Initial phase of the objective-changing sequence, in which the first detection objective begins to swing away from the active imaging position. (**d**) Intermediate phase of the swing motion during objective exchange. (**e**) Final phase of the exchange, in which the swing motion is completed and the second detection objective is positioned in the active imaging location. Abbreviations: 1, first detection objective (XLPLN10XSVMP oil; NA = 0.6; WD = 8 mm; Evident); 2, second detection objective (CFI90 20XC Glyc; NA = 1.00; Nikon); io_1_, first illumination objective; io_2_, second illumination objective; ioc, immersion oil chamber.

A detection objective changer (DOC) enables flexible switching of detection objectives during specimen examination, with repeatability better than 3 µm; residual positional variability is corrected by system software (Figure 3).

In this regard, it is important to understand that, due to the fixed positioning of the illumination objectives in the LSTM technology and ClearScope (io_1_ and io_2_ in Figure 3a), it is not feasible to incorporate a standard objective turret as used in epi-fluorescence microscopes. This constraint is inherent to the optical design of the LSTM technology and ClearScope.

**Figure 4:**
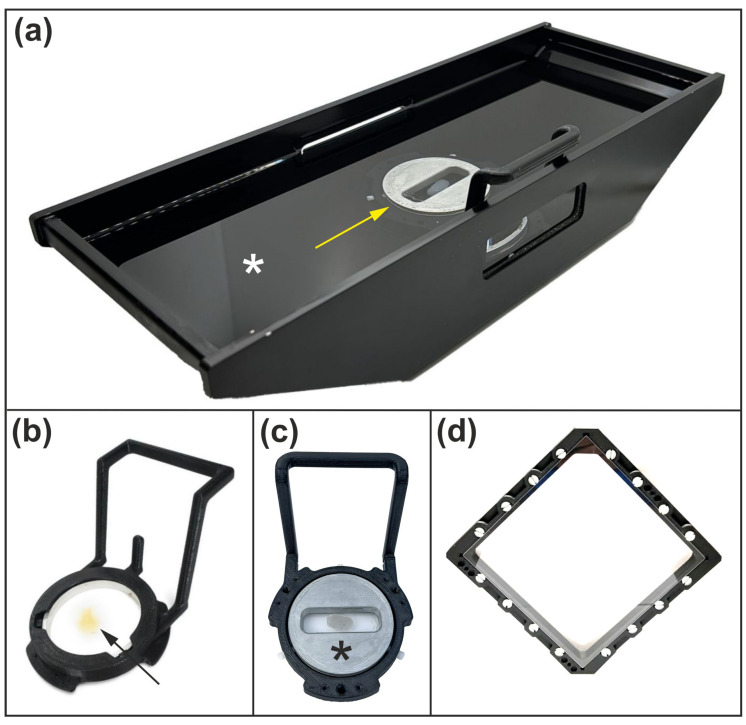
Specimen holding assemblies developed for the ClearScope microscope. (**a**) Immersion oil chamber (to be mounted on a motorized stage insert), showing the rectangular aluminum tub filled with immersion oil (white asterisk in (**a**)) and the centrally positioned specimen chamber (yellow arrow in (**a**)) secured by a magnetic coupler. (**b**) Standard specimen chamber (black arrow in (**b**)) and chamber holder, illustrating the removable chamber design that accommodates whole mouse or rat brains between two circular coverslips separated by adjustable silicon gaskets. (**c**) Low-volume specimen chamber (black asterisk in (**c**)) inserted into the chamber holder for imaging smaller specimens. (**d**) Large specimen chamber for large specimens, showing the symmetric design with extended lateral dimensions that enables imaging from either side of the specimen. Details are provided in the text.

Specimens are mounted in a rectangular aluminum immersion-oil chamber mounted on a stage insert (Figure 4a).

A central specimen chamber (Figure 4b) is secured by a magnetic coupler and chamber holder and can accommodate intact mouse or rat brains. The chamber consists of two circular coverslips separated by silicone gaskets that provide adjustable height, all housed within an aluminum ring with a threaded internal collar to ensure reliable sealing.

For smaller specimens, a low-volume insert can be used (Figure 4c), reducing the volume of immersion medium required by the tissue-clearing protocol to approximately 3–5 mL. For larger specimens, a dedicated large-volume chamber was developed with internal dimensions of 144 × 144 × 8 mm. This chamber features a symmetric design that enables imaging from either side (Figure 4d). Vacuum grease channels are used to seal the specimen windows, and the assembly is secured using the same magnetic/mechanical detent mechanism employed by other holders.

Note: The immersion oil chamber is available in multiple sizes; however, the largest configuration (shown in Figure 4a) requires approximately 0.5 L of refractive index (RI)-matching liquid to fill. The required volume depends on the thickness of the loaded specimen chamber. Owing to the ClearScope microscope’s ability to compensate for RI mismatch (described below), a single RI-matching medium can be used across a broad range of clearing media, spanning RIs from 1.33 (water) to 1.56 (iDISCO [9]), without the need to exchange the immersion medium.

Image acquisition software was developed as a fully integrated Windows 11 (64-bit) desktop application written in C++. It uses MBF Bioscience core software libraries and object-oriented design methodologies. Development was carried out using Microsoft Visual Studio Professional 2019 (Microsoft, Redmont, WA, USA), with extensive profiling and optimization for memory, central processing unit (CPU) and graphics processing unit (GPU) performance. Multi-threaded processing is employed throughout. Considerable effort was devoted to user interface design, documentation and usability testing, resulting in a fully documented user guide and support tools.

**Figure 5:**
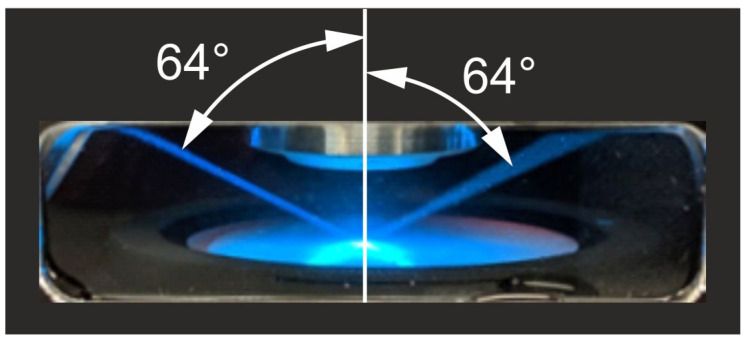
Visualization of the two light sheets of the ClearScope microscope, viewed through the window in the immersion oil chamber (yellow arrow in Figure 3a). Imaging is performed along an “illumination line” (see Figure 2b–d) at the position where the thinnest parts of both light sheets overlap (minimum thickness 3.2 µm).

The software synchronizes camera exposure, galvo scanning and electrically tunable lens focus to maintain the thinnest, most focused portion of the light sheets during acquisition (Figure 5).

Two-axis scanning synchronization was optimized using polynomial regression based on seven calibration points across the field of view, accounting for nonlinearities in electrically tunable lens behavior and enabling the use of larger objectives and cameras. Intelligent Refractive Index Compensation (IRIC) was implemented to automatically adjust light-sheet position and focus as a function of tissue depth for each color channel, enabling imaging of specimens cleared using a wide range of protocols [5,6,7,8,9,10,11,12,13]. For large and heterogeneous samples, a 3D IRIC mapping approach was developed that incorporates correction data from multiple XY locations.

To prevent acquisition failure during long unattended imaging sessions, the software includes a disk-space verification function that estimates required storage and confirms availability prior to acquisition. Post-processing tools include flat-field correction and blending algorithms for seamless stitching of multiple fields of view, a rolling-ball filter for uneven background correction and an unsharp mask filter for image enhancement. Illumination distortions are modeled as a linear gain function combined with an additive term; distortion fields are estimated retrospectively using regularized energy minimization [16] and reversed to generate corrected images. Linear blending is applied in overlapping regions to improve montage quality.

Image acquisition speed was optimized by overlapping software operations with mechanical movements, reducing exposure times, employing a Prime BSI Express camera (2048 × 2048 pixels; rolling shutter; Teledyne Photometrics, Tucson, AZ, USA) and improving electrically tunable lens focus performance. The fastest achieved exposure time was 45.9 ms per plane using a rolling shutter.

An intuitive interface was developed for configuration and operation of the detection objective changer and for integration with several single-mode fiber laser systems. Comparative testing showed that single-mode fiber lasers produced thinner light sheets and higher axial resolution than multi-mode systems.

Automatic tissue detection, combined with motorized stage movement and the DOC, enables low-resolution scanning followed by targeted high-resolution imaging. A Tissue Scanning Workflow guides users through multi-resolution acquisition. After low-resolution imaging, users define regions of interest on preview images, switch detection objectives and acquire high-resolution data from selected regions. Post-acquisition tools such as 3D stitching and flat-field correction can then be applied.

The image quality of the ClearScope microscope was quantitatively assessed by estimating the point spread function (PSF). To this end, 36 three-dimensional images of the same field of view were acquired from a slide containing fluorescent microspheres (0.5 µm diameter; FocalCheck™ Fluorescence Microscope Test Slide #5; RI = 1.515; ThermoFisher Scientific, Waltham, MA, USA). Imaging was performed in an immersion medium with an RI of 1.4587 using an XLPLN10XSVMP objective (10×/0.6 NA, infinity-corrected; working distance = 8 mm; Evident) and IRIC. Images were acquired using a 561 nm excitation laser and a Prime BSI Express camera (2048 × 2048 pixels; rolling shutter; Teledyne Photometrics).

Note: (i) Because of the practical trade-off between using a thinner light sheet and maintaining suitable camera exposure times across different samples, PSF measurements were acquired using a light sheet with a thickness of approximately 6 µm. (ii) The relationship between light-sheet thickness and axial FWHM in ClearScope differs from that in standard light-sheet microscopes because the illumination enters the sample at an oblique angle (cf. Figure 3c in [14]). Given a light-sheet thickness of 6 µm and an illumination angle of θ = 64° (cf. Figure 5), the effective planar illumination thickness during PSF imaging was 6 µm/sin 64° ≈ 6.5 µm. Accordingly, the PSF values reported here represent conservative, real-world measurements.

For this optical configuration, the axial resolution (FWHM of the axial PSF) is approximately 6.5 µm. Images were acquired with an axial sampling interval of 1.5 µm (≈4.3 samples per axial resolution element), satisfying the Nyquist sampling criterion, which requires at least two samples per resolvable feature equivalently, sampling at twice the highest spatial frequency of the axial PSF [17]. All 36 fields of view were registered using BigStitcher v 1.1.2 [18] and subsequently summed in Fiji v 1.54m [19] to improve the signal-to-noise ratio. Full width at half maximum (FWHM) measurements were obtained at the center and corners of the summed field of view using the intensity profiling tool in Fiji [19]. It should be noted that better axial resolution can be achieved by using thinner light sheets, since the field of view is not limited by the light-sheet thickness in this configuration. Since an ETL is used to scan the thinnest part of the light sheet, thinner light sheets could be employed in principle to further improve axial resolution.

Alignment of the different image channels was also assessed using a slide containing fluorescent microspheres (diameter 1.5 µm; Focal Check Fluorescence Microscope Test Slide #1; ThermoFisher Scientific). Imaging was performed using the same 10× objective with 405 nm, 488 nm, 561 nm and 640 nm excitation lasers.

Product validation and usability studies were conducted using surplus material from ongoing research projects in our laboratories. The surgery was done in live patients who had epilepsy for temporal lobe epilepsy treatment following NYU Langone Health IRB [20]. Additional mouse brain samples were provided by Binaree, Inc. (Daegu, Republic of Korea). No specific animal experiments were performed as part of system development, and no IACUC approval was required. Imaging and analysis were performed at the MBF Bioscience office.

## 3. Results

### 3.1. Assessment of ClearScope Image Quality

**Figure 6:**
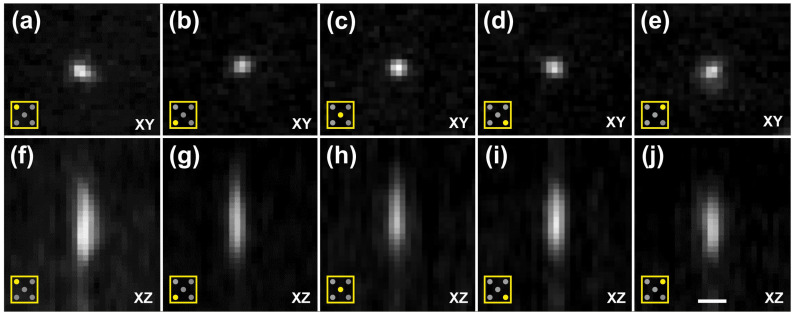
Estimation of the point spread function (PSF) of the ClearScope microscope. All panels show cross-sections generated using the Orthogonal Views function in Fiji [19] (22 images per stack; pixel size 0.589 µm in XY; axial spacing 1.5 µm; single XY plane shown). Images were acquired from a slide containing fluorescent microspheres (diameter 0.5 µm; Focal Check Fluorescence Microscope Test Slide #5; ThermoFisher Scientific) using a ClearScope equipped with an XLPLN10XSVMP objective (10×/0.6 NA ∞, WD = 8 mm; Evident), a 561 nm excitation laser and a Prime BSI Express camera (Teledyne Photometrics). (**a**–**e**) XY cross-sections of image stacks, showing representative regions across the field of view, including the upper-left (**a**), lower-left (**b**), center (**c**), lower-right (**d**) and upper-right (**e**) quarters of the field of view, as indicated by the pictograms in the lower-left corner of each panel. (**f**–**j**) Corresponding XZ cross-sections of the same image stacks, shown for the upper-left (**f**), lower-left (**g**), center (**h**), lower-right (**i**) and upper-right (**j**) regions of the field of view, enabling assessment of axial resolution and light-sheet thickness across the imaging area. These image stacks were used to determine the PSF of ClearScope. The scale bar in (**j**) represents 3 µm in (**a**–**e**) and 2.8 µm in (**f**–**j**).

Figure 6 shows cross-sections of image stacks generated using Orthogonal Views in Fiji [19] of a slide containing fluorescent microspheres (diameter, 0.5 µm; Focal Check Fluorescence Microscope Test Slide #5; ThermoFisher Scientific).

The axial FWHM ranged from 5.45 µm to 6.37 µm, while the lateral FWHM ranged from 0.34 µm to 0.37 µm (Table 1; data were derived from averaged datasets).

These measurements demonstrate that ClearScope achieves subcellular-resolution imaging (with subcellular resolution determined by the lateral performance of the system)—and, in lateral dimensions, even sub-micron resolution—using a 10× objective across the full field of view and throughout specimens with effectively unlimited lateral (XY) extent, constrained only by the objective’s working distance.

**Figure 7:**
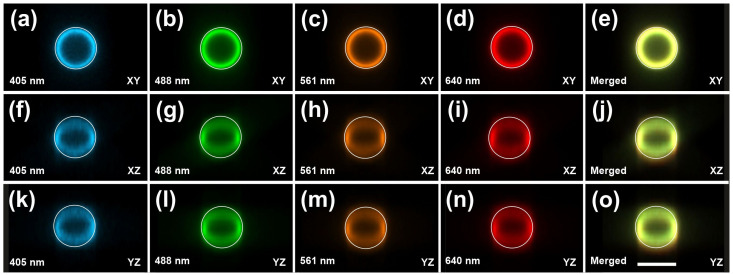
Assessment of multichannel image quality and alignment of the ClearScope microscope. Images were acquired from a slide containing fluorescent microspheres (diameter 15 µm; Focal Check Fluorescence Microscope Test Slide #1; ThermoFisher Scientific) using a ClearScope equipped with an XLPLN10XSVMP objective (10×/0.6 NA ∞, WD = 8 mm; Evident), a Prime BSI Express camera (Teledyne Photometrics) and excitation lasers as indicated in the panels. All panels show cross-sections generated using the Slice Viewer in the 3D environment of the Neurolucida 360 software v 2025.1 (MBF Bioscience) from image stacks consisting of 25 images (pixel size 0.589 µm in XY; axial spacing 1.5 µm; single XY plane shown). (**a**–**e**) Channel-specific XY cross-sections of fluorescent microspheres emitting at different wavelengths, acquired using 405 nm (**a**), 488 nm (**b**), 561 nm (**c**), and 640 nm (**d**) excitation lasers, along with a merged multichannel XY view (**e**). (**f**–**o**) Corresponding channel-specific XZ cross-sections (**f**–**i**) and merged XZ view (**j**), as well as channel-specific YZ cross-sections (**k**–**n**) and merged YZ view (**o**), enabling assessment of isotropy and multichannel alignment. The white rings overlaid in each panel have identical diameters and were added during figure preparation for visual reference. When viewed isotropically, the beads appear spherical, reflecting the very thin light sheets of ClearScope (minimum thickness 3.2 µm) and demonstrating precise alignment across all color channels. The scale bar in (**o**) represents 15 µm.

When visualized isotropically, the beads appeared spherical, reflecting the thin light sheets generated by ClearScope (minimum thickness 3.2 µm). No misalignment between color channels was observed, indicating precise multichannel registration (Figure 7).

### 3.2. Imaging of Biological Specimens

A wide range of biological specimens was imaged with ClearScope to demonstrate its performance under realistic experimental conditions.

#### 3.2.1. Representative Example of Imaging an Entire, Intact Mouse Brain Using ClearScope

**Figure 8:**
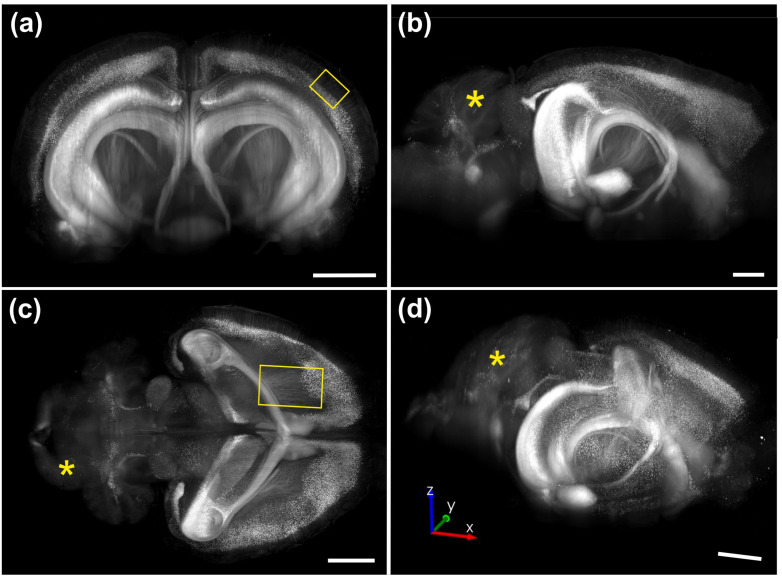
Whole-brain imaging of an intact mouse brain using a ClearScope microscope. (**a**–**d**) Maximum-intensity projections of portions of a 3D image of an entire, intact brain from a transgenic mouse expressing eGFP under control of the Thy-1 promoter, shown in coronal (**a**), sagittal (**b**), horizontal (**c**) and oblique (arbitrary) (**d**) views. Images were acquired using ClearScope equipped with an MBF Bioscience-modified UPLFLN4XPH objective (4×/0.13 NA; WD = 17 mm; Evident), a 488 nm excitation laser, a ZET405/488/561/640mv2 quad-band emission filter (Chroma Technologies, Bellow Falls, VT, USA), and a Prime BSI Express camera (Teledyne Photometrics). The total acquisition duration was 8 h 32 min. The yellow rectangles in (**a**,**c**) are explained in the text, as are the yellow asterisks * (**b**–**d**). Each scale bar represents 2 mm. Note: the total acquisition time for this whole-brain dataset was 8 h 32 m, underscoring that the ClearScope is capable of fully unattended (‘lights-out’) operation.

Whole-brain imaging was performed on an intact brain from a transgenic mouse expressing eGFP under control of the Thy-1 promoter. The brain specimen was provided by Binaree, Inc. (Daegu, Republic of Korea) and cleared using the Binaree Tissue Clearing protocol [13]. Images were acquired using an MBF Bioscience-modified UPLFLN4XPH objective (4×/0.13 NA; modified WD = 12 mm; Evident) (Figure 8).

The weak eGFP signal observed in the cerebellum (asterisks in Figure 8b–d) was expected [21]. The absence of vertical or horizontal tiling artifacts demonstrates that the 3D dataset can be viewed and analyzed from arbitrary orientations, an essential requirement for unbiased and reproducible digital neuron tracing in three dimensions.

#### 3.2.2. Representative Example of Imaging of Neuronal Structures in Mouse Brain Tissue Using ClearScope

**Figure 9:**
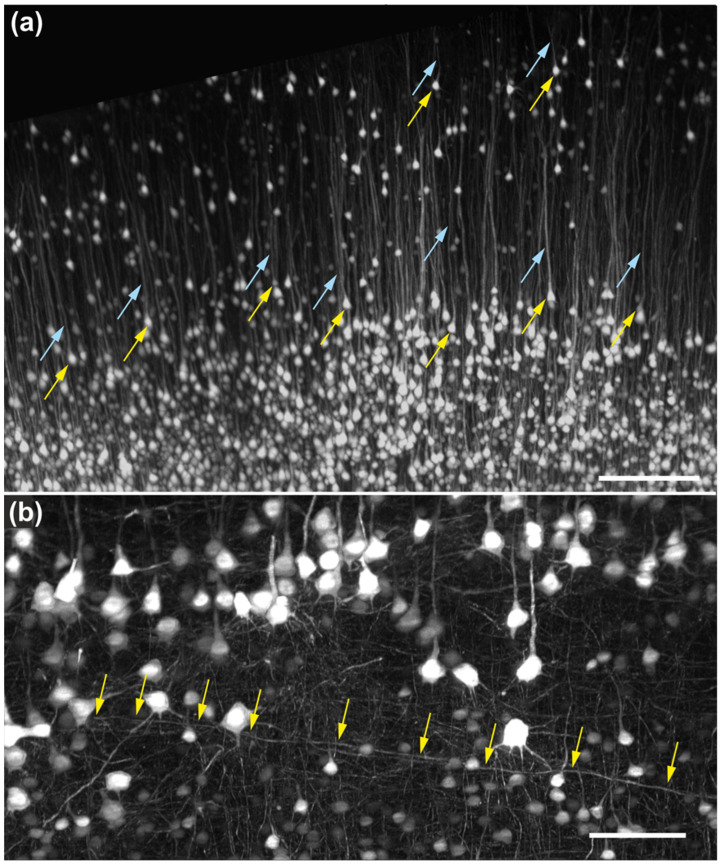
High-resolution imaging of neuronal structures in mouse brain tissue using the ClearScope microscope. (**a**) High-magnification region-of-interest acquisition of the area indicated by the yellow rectangle in Figure 8a, showing a portion of an intact brain from a transgenic mouse expressing eGFP under control of the Thy-1 promoter. Images were acquired using a ClearScope equipped with an XLPLN10XSVMP objective (10×/0.6 NA ∞, WD = 8 mm; Evident), a 488 nm excitation laser, a ZET405/488/561/640mv2 quad-band emission filter (Chroma) and a Prime BSI Express camera (Teledyne Photometrics). The total acquisition duration 32 min. Yellow arrows indicate the cell bodies of individual neurons, and light blue arrows indicate the apical dendrites of the same neurons. (**b**) Higher-magnification view of the cerebral cortex from the same brain, acquired using the same imaging configuration as in (**a**) and deconvolved using the Neuro Deblur software v2025.1 (MBF Bioscience). Arrows indicate a neuronal process passing tangentially through the cortex that could be traced over a distance of approximately 650 µm. The scale bar in (**a**) represents 250 µm and the scale bar in (**b**) 100 µm.

Higher-magnification region-of-interest acquisitions of the same mouse brain shown in Figure 8 were obtained using an XLPLN10XSVMP objective (10×/0.6 NA; Evident) (Figure 9).

These images enabled unequivocal identification of neuronal cell bodies in the cerebral cortex as well as visualization of apical dendrites (Figure 9a). Individual neuronal processes could be traced over distances of several hundred micrometers, including tangentially oriented fibers traversing the cortex (Figure 9b). In subcortical white matter, individual axonal processes could likewise be resolved and traced.

#### 3.2.3. Representative Example of Imaging and Reconstruction of Neuronal Structures in Mouse and Human Brain Tissue Using the ClearScope System

**Figure 10:**
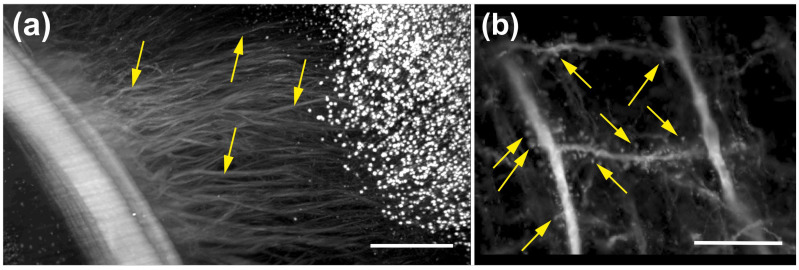
High-resolution imaging of neuronal structures in mouse brain tissue using the ClearScope microscope. (**a**) High-magnification view of the subcortical white matter region indicated by the yellow rectangle in Figure 8c, acquired from an intact brain of a transgenic mouse expressing eGFP under control of the Thy-1 promoter (Thy1-eGFP). Images were acquired using ClearScope equipped with an MBF Bioscience-modified UPLFLN4XPH objective (4×/0.13 NA; modified WD = 17 mm (Olympus, Tokyo, Japan); a 488 nm excitation laser, a ZET405/488/561/640mv2 quad-band emission filter (Chroma Technologies), and a Prime BSI Express camera (Teledyne Photometrics). Arrows indicate individual neuronal processes that can be traced. (**b**) High-magnification maximum-intensity projection of a portion of a 3D image of a Thy1-eGFP mouse brain cleared using CLARITY [5,6,7]. Imaging was performed using ClearScope equipped with a CFI90 20XC Glyc objective (20×/1.0 NA ∞, WD = 8.2 mm; Nikon), a 488 nm excitation laser, a ZET405/488/561/640mv2 quad-band emission filter (Chroma Technologies) and an Orca-Flash camera operated in V3 mode (Hamamatsu Photonics, Hamamatsu City, Japan). The total acquisition duration was 10 min. Arrows indicate quantifiable dendritic spines of cortical neurons. The scale bar in (**a**) represents 500 µm and the scale bar in (**b**) 100 µm.

At even higher magnification, dendritic spines of cortical neurons were clearly resolved in mouse brain tissue using a CFI90 20XC Glyc objective (20×/1.0 NA; Nikon; Figure 10a,b).

**Figure 11:**
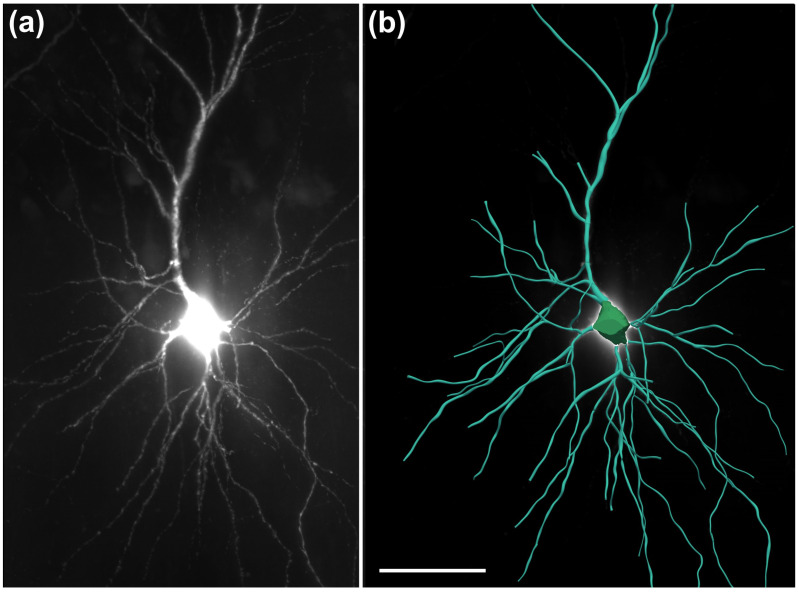
High-resolution imaging and reconstruction of neuronal structures in human brain tissue using the ClearScope microscope. (**a**) 3D image of a portion of a human hippocampal CA1 pyramidal neuron obtained from a patient undergoing surgical treatment for drug-resistant epilepsy [20]. Neurons were filled with biocytin during patch-clamp recordings, the tissue was cleared using CUBIC [8], and neurons were subsequently labeled with streptavidin conjugated to Alexa 555 (ThermoFisher Scientific). Imaging was performed using a ClearScope equipped with a CFI90 20XC Glyc objective (Nikon), a 561 nm excitation laser, a ZET405/488/561/640mv2 quad-band emission filter (Chroma Technologies) and an Orca-Flash camera operated in V3 mode (Hamamatsu Photonics). The total acquisition duration was 20 min. (**b**) Digital reconstruction of the neuron shown in (a), generated for quantitative analysis using the automated 3D neuron reconstruction software Neurolucida 360 (MBF Bioscience). The scale bar in (**b**) represents 100 µm in (**a**,**b**).

The same objective was used to image ex vivo human neurons that had been filled with biocytin during patch-clamp recordings and subsequently stained with streptavidin conjugated to Alexa 555. (ThermoFisher Scientific); the neurons were in acute slices derived from surgically resected human hippocampal CA1 (Figure 11) [20].

These data were of sufficient quality to allow for automated 3D reconstruction and quantitative analysis using the Neurolucida 360 software (MBF Bioscience) (Figure 11b).

#### 3.2.4. Representative Example of Imaging, Visualization and Digital Reconstruction of Vascular Networks in Mouse Brain Tissue Using ClearScope

**Figure 12:**
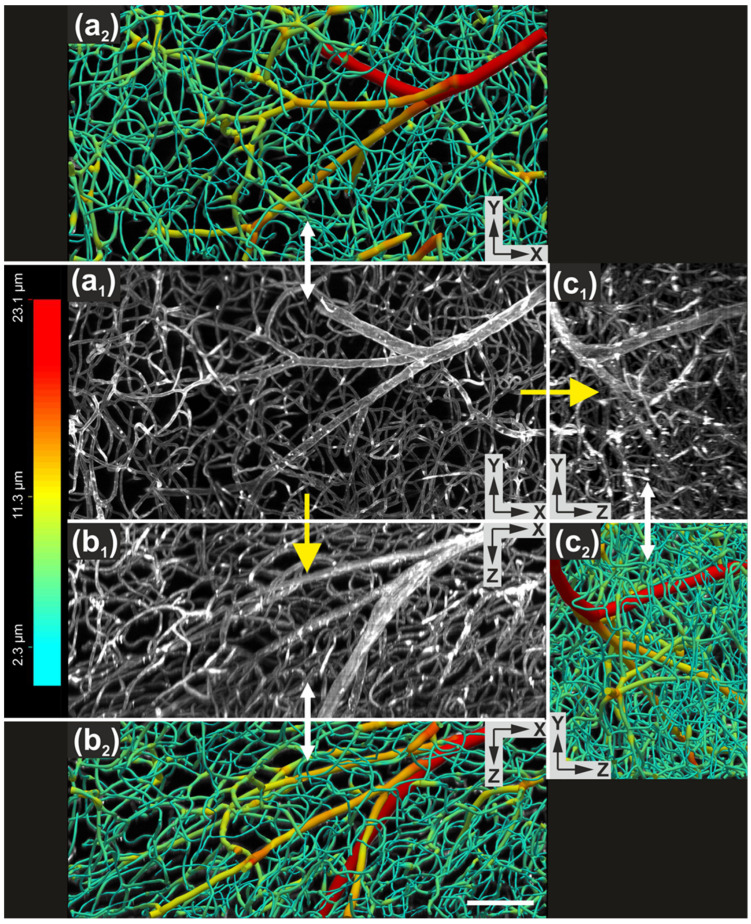
Imaging, visualization and digital reconstruction of vascular networks in mouse brain tissue using the ClearScope microscope. (**a_1_**–**c_1_**) Maximum-intensity projections of a portion of a 3D image of an entire, intact mouse brain with blood vessels labeled using *Lycopersicon esculentum* (tomato) lectin conjugated to DyLight 649 (ThermoFisher Scientific), shown in XY (**a_1_**), XZ (**b_1_**) and YZ (**c_1_**) views. Images were acquired using ClearScope equipped with an XLPLN10XSVMP objective (10×/0.6 NA ∞, WD = 8 mm; Evident), a 640 nm excitation laser, a ZET405/488/561/640mv2 quad-band emission filter (Chroma Technologies) and a Prime BSI Express camera (Teledyne Photometrics). The total acquisition duration was 18 min. The brain specimen was provided by Binaree, Inc. and cleared using the Binaree Rapid Tissue Clearing protocol [13]. (**a_2_**–**c_2_**) Digital reconstructions of the vascular structures shown in (**a_1_**–**c_1_**), generated for quantitative analysis using the automated 3D reconstruction software Neurolucida 360 v 2025.1 (MBF Bioscience). Vessel diameters are color-coded as indicated. The scale bar in (**b_2_**) represents 100 µm.

**Figure 13:**
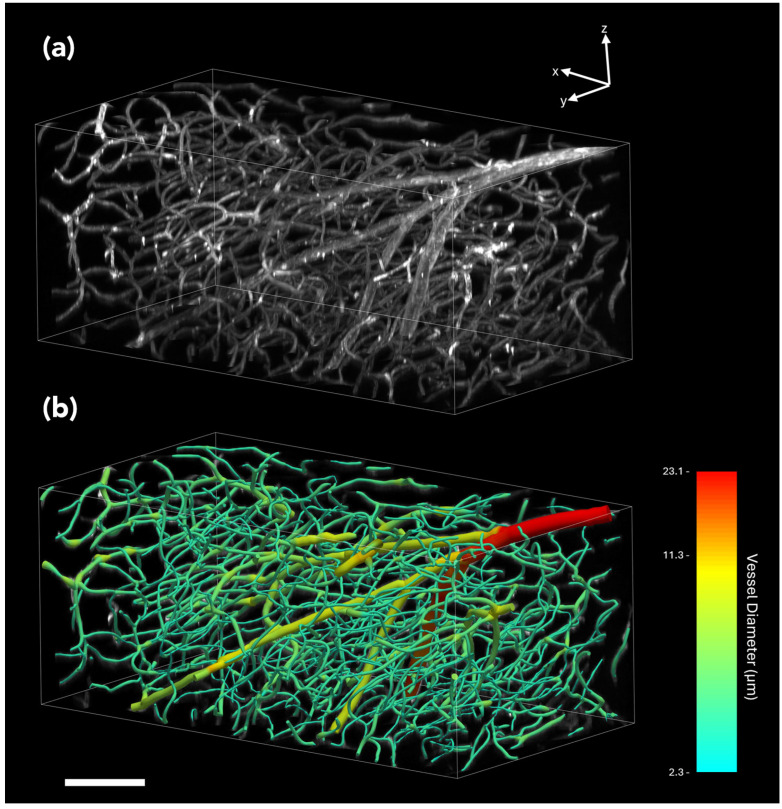
3D visualization and digital reconstruction of vascular networks in mouse brain tissue using the ClearScope microscope. (**a**) 3D rendering of a 3D image of an entire, intact mouse brain with blood vessels labeled using *Lycopersicon esculentum* (tomato) lectin conjugated to DyLight 649 (ThermoFisher Scientific). The dataset corresponds to the maximum-intensity projections shown in Figure 12a_1_–c_1_ and was acquired using a ClearScope microscope. (**b**) Digital reconstruction of the vascular network shown in (**a**), generated for quantitative analysis using the automated 3D reconstruction software Neurolucida 360 v 2025.1 (MBF Bioscience). Vessel diameters are color-coded as indicated. Scale bar in (**b**) represents 100 µm.

Vascular imaging was demonstrated in whole mouse brains with blood vessels labeled using Lycopersicon Esculentum (Tomato) Lectin conjugated to DyLight 649 (ThermoFisher Scientific). Imaging with a 10× objective yielded high-contrast 3D datasets from which vascular networks could be digitally reconstructed and quantitatively analyzed using automated reconstruction software (Figure 12 and Figure 13).

### 3.3. Registration of 3D Mouse Brain Images Acquired with ClearScope to the Allen Mouse Brain Common Coordinate Framework (CCFv3)

**Figure 14:**
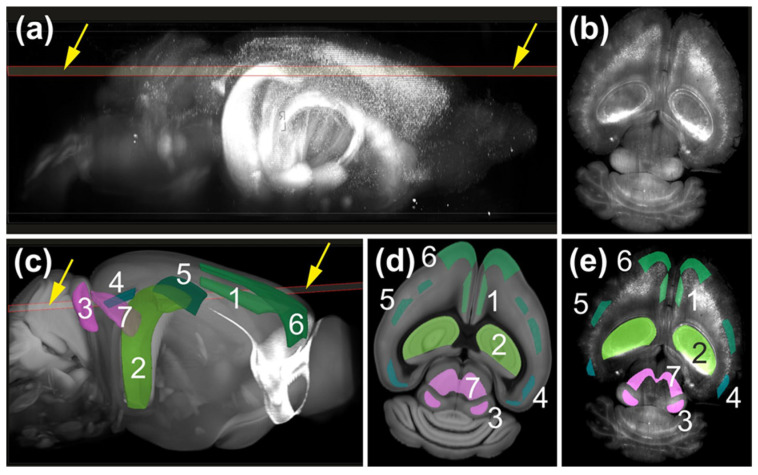
Registration of 3D mouse brain images acquired with the ClearScope system to the Allen Mouse Brain Common Coordinate Framework (CCFv3) [22,23]. (**a**) Selection of a virtual section (yellow arrows in (**a**)) through the 3D image of an entire, intact brain from a transgenic mouse expressing eGFP under control of the Thy-1 promoter, acquired using a ClearScope microscope (dataset shown in Figure 8b). (**b**) The selected virtual brain section shown in (**a**). (**c**) Automatic registration of the volumetric brain image and the selected virtual brain section (arrows) to the CCFv3 [22,23] using the NeuroInfo software v 2025.1 (MBF Bioscience). Representative anatomical regions defined by the CCFv3 are indicated: (1) anterior cingulate area, dorsal part, layer 5; (2) hippocampal field CA1; (3) inferior colliculus, central nucleus; (4) lateral visual area, layer 5; (5) primary somatosensory area, barrel field, layer 4; (6) secondary motor area, layers 2–3; (7) superior colliculus, motor-related, intermediate white layer. (**d**) Virtual section through the CCFv3 showing the anatomical regions corresponding to those identified in (**c**), representing the best match to the virtual mouse brain section shown in (**a**,**c**). (**e**) Automatic delineation of the anatomical regions identified in (**c**,**d**), overlaid on the virtual mouse brain section selected in (**a**,**c**). This workflow enables automated identification of anatomical regions in low-magnification whole-brain datasets acquired with ClearScope, supporting selective high-magnification imaging and subsequent automated or computer-assisted quantitative analysis.

The high image quality obtained from intact mouse brains enabled virtual sectioning of the 3D datasets and registration with the Allen Mouse Brain Common Coordinate Framework (CCFv3) [22,23] (Figure 14).

This capability supports anatomically informed quantitative analyses and facilitates workflows that combine rapid low-magnification screening with subsequent high-resolution imaging of selected anatomical regions of interest. Such approaches can be integrated with systematic random sampling strategies based on design-based stereology [24], enabling increased imaging efficiency, reduced data volume and comprehensive documentation of analyzed regions.

## 4. Discussion

There is a growing need for microscopy systems capable of imaging intact organs at high resolution. The initial application domain of the ClearScope system is neuroscience research. The representative photomicrographs shown in Figure 9, Figure 10, Figure 11 and Figure 12 demonstrate the substantial potential of ClearScope for connectomics research. In the example shown in Figure 9, total sample processing time, including fixation and clearing, was approximately 10 days, while image acquisition required 8 h. Beyond applications in neuroscience focused on the central nervous system, ClearScope is also well suited for studies in systems biology aimed at understanding innervation patterns in peripheral organs, cancer research investigating angiogenesis in tumor-affected tissues, and the analysis of organoids.

Although no commercially available light-sheet microscope currently available matches the combination of image quality and unconstrained lateral imaging capability of ClearScope, numerous commercial light-sheet microscopes are on the market. Table 2 (modified from [25]) summarizes and compares key technical specifications—including detection objective numerical aperture, axial resolution, supported refractive index range, maximum imaging depth and maximum lateral specimen size—of commercially available light-sheet microscopes, as well as the recently developed hybrid open-top light-sheet (Hybrid OTLS) microscope [25].

**Figure 15:**
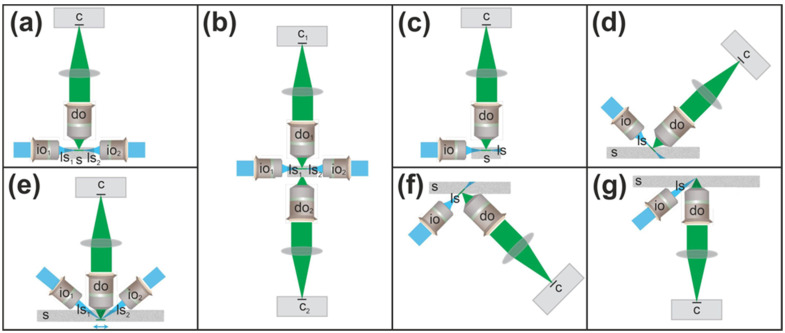
Schematic comparison of imaging principles used in light-sheet microscopy systems summarized in Table 2. (**a**–**g**) Schematic representations of the optical geometries and imaging principles of different light-sheet microscope configurations, illustrating the relative orientations of illumination objectives, detection objectives, light sheets and specimens. The diagrams highlight key differences in illumination and detection strategies that underlie the performance characteristics summarized in Table 2. Abbreviations: c, camera; do, detection objective; io, illumination objective; ls, light sheet; s, specimen. The panels were modified from [14], published under the CC BY 4.0 license URL: https://creativecommons.org/licenses/by/4.0/ (accessed on 17 January 2025). Details are provided in the text.

Figure 15 illustrates the imaging principles underlying the light-sheet microscope architectures summarized in Table 2.

The classical SPIM design, which employs one or more light sheets orthogonal to the detection path [26,27] (Figure 15a), is implemented in systems such as the Blaze (Miltenyi Biotec), Alpha3 (PhaseView) and Lightsheet 7 (Zeiss). However, this architecture inherently constrains lateral imaging extent (Table 2). The MuVi SPIM CS (Bruker) uses a modified SPIM configuration with two illumination paths and two detection objectives (Figure 15b) but retains the same fundamental lateral limitations.

Several systems—including SmartSPIM and MegaSPIM (both Lifecanvas), ct-dSPIM (ASI), CTLS and AxL Cleared Tissue (both Intelligent Imaging Innovations; III)—are based on axially swept light-sheet microscopy (ASLM) technology [28] (Figure 15c,d). ASLM employs axial sweeping of a light sheet across the field of view within a focal plane, with fluorescence collected using a synchronized rolling shutter. In contrast, LSTM adopts a fundamentally different strategy: the light sheet is scanned simultaneously along two orthogonal axes—along the direction of light-sheet propagation and perpendicular to it—in synchrony with rolling-shutter readout by a CMOS camera. This simultaneous two-axis scanning enables rapid scanning of a thin excitation line, defined by the intersection of the thinnest region of an oblique light sheet with the detection focal plane, within the detection focal plane. In ALSM systems, the angle between the long axis of the light sheet and the detection objective is restricted to 90°, as smaller angles are precluded by the LSTM patent [29]. When the detection objective is oriented perpendicular to the specimen surface (Figure 15c), as in SmartSPIM (Lifecanvas) and AxL Cleared Tissue (III), lateral imaging extent is constrained. Alternatively, when the detection objective is oriented at an angle (e.g., 45°; Figure 15d), as in MegaSPIM (Lifecanvas) and ct-dSPIM (ASI), the maximum achievable imaging depth is limited.

Lateral imaging unconstrained by the configuration of the illumination and detection objectives is only possible with ClearScope (Figure 15e), ct-dSPIM (ASI) and Hybrid OTLS [25] (Figure 15f,g). Compared to ct-dSPIM, ClearScope supports low-magnification (4× and 5×) detection objectives in addition to high-magnification objectives (10×, 20×, 24× and 27×) and provides substantially greater imaging depth. Specifically, when using a 16× detection objective, the maximum penetration depth exceeds that of ct-dSPIM (ASI) by more than a factor of two (12 mm vs. 5 mm), and when using a 24× detection objective, by a factor of five (10 mm vs. 2 mm).

MegaSPIM (Lifecanvas) supports a maximum specimen lateral size of 200 mm × 200 mm, enabling imaging of large, cleared tissue slabs from human and non-human primate postmortem brains. However, because the detection objective is oriented at 45° relative to the specimen surface, the maximum achievable imaging depth is limited to WD × sin(45°) = WD × 0.707, where WD is the working distance of the detection objective.

Hybrid OTLS [25] is the only open-top light-sheet microscope included in this comparison. It employs a conventional orthogonal architecture for low-magnification imaging (Figure 15f) and a non-orthogonal architecture for high-magnification imaging (Figure 15g). The latter configuration prevents commercialization, as angles smaller than 90° between the light-sheet and detection axes are protected by the LSTM patent [29]. The developers of Hybrid OTLS identified five key requirements for next-generation light-sheet microscopes: (i) user-friendly mounting of multiple specimens with standard holders; (ii) compatibility with all major tissue-clearing protocols; (iii) no fundamental limits on lateral specimen size; (iv) large imaging depth for intact mouse organs and thick tissue slabs; and (v) broad multi-scale imaging capabilities for time- and data-efficient workflows [25]. The ClearScope system meets all of these requirements.

As published, Hybrid OTLS [25] has several disadvantages compared to ClearScope: (i) a considerably more complex illumination path; (ii) availability of only two detection objectives (2× and 24×), both with lower axial resolution than the ClearScope objectives (Table 2); (iii) no ability to change detection objectives according to experimental needs; and (iv) a low-magnification (2×) detection objective oriented at 45° relative to the specimen surface, limiting maximum imaging depth to 10 mm, compared to 12 mm achievable with ClearScope’s 4× objective.

It should be noted that the authors of [25] stated that synchronizing the two light sheets in LSTM (and, thus, in the ClearScope system) to a rolling shutter would result in inefficient light collection and fundamental losses introduced by the LSTM scanning strategy (Supplementary Note 1 in [25]). This assertion overlooks the well-established advantages of line-scan imaging strategies in light-sheet microscopy, which are central to both ASLM [27] and LSTM implementations [14].

In addition to commercial systems, OpenSPIM represents an open-source SPIM option [30,31]. Although a functional SPIM system can be constructed from commercial components and 3D-printed or custom-machined parts, OpenSPIM systems are limited to smaller specimens, rely on low-precision positioning devices and require substantial mechanical expertise for assembly and alignment. Imaging speed is slow, and neither the original OpenSPIM [30] nor X-OpenSPIM [31] provides unconstrained lateral imaging.

Another open-source light-sheet microscope is mesoSPIM [32]. While it features a travel range of 50 × 50 × 100 mm, this does not necessarily mean that specimens of this size can be fully imaged. Similar to OpenSPIM [30] and X-OpenSPIM [31], mesoSPIM [32] does not support lateral imaging that is unconstrained by the configuration of the illumination and detection objectives.

Early adopters have already used ClearScope systems to publish high-impact biomedical research [33,34,35,36]. For example, in one study [33], ketamine was repeatedly administered to mice, followed by whole-brain imaging of dopamine neurons using a ClearScope microscope equipped with 4× and 20× objectives. The cleared samples measured approximately 15 × 20 mm laterally and 9 mm in depth. The authors identified dose-dependent changes in dopamine neuron distributions and altered innervation patterns across multiple brain regions. In another study [36], ClearScope was used to image entire centimeter-thick human brain slabs that had been cleared and labeled using the CleLight protocol. The system enabled high-resolution 3D imaging of archival FFPE (formalin-fixed paraffin-embedded) brain tissue blocks from Alzheimer’s disease patients and non-diseased donors, allowing researchers to map blood vessels, amyloid-β plaques, phosphorylated tau, microglia, neurons, and other pathological features throughout large tissue volumes. According to Table 2, these studies would not have been feasible with any other commercially available light-sheet microscope without physically sectioning the brain, which would compromise connectivity reconstruction. While Hybrid OTLS could theoretically have been used, it is not commercially available.

The development of ClearScope directly addresses two key requests articulated by experts in the field [37]: (i) prioritizing practicality and applicability to biological and biomedical research over maximal optimization of isolated technical specifications (such as the numerical aperture that can be covered in LSM [38,39]), and (ii) enabling smart imaging strategies that survey specimens at low magnification and autonomously transition to high-magnification imaging only in regions of interest. Such approaches substantially reduce dataset sizes compared to uniform high-magnification scanning.

One limitation of ClearScope is that LSTM requires higher illumination intensity than traditional light-sheet microscopy, necessitating more powerful lasers. This requirement is justified by the resulting image quality. A related limitation is that the illumination optics are optimized for high-magnification objectives (up to 25×), precluding the use of very low magnification objectives (e.g., 2×).

Another limitation is the absence of an application programming interface (API). In this regard, it was stated in a recent review [37] on LSM that it is considered essential in the neuroscience community that manufacturers of commercial microscopy systems offer interfaces for microscope control, imaging and image analysis using open-source software (e.g., Micro-Manager [40], Pycro-Manager [41], AutoScanJ [42], ImSwitch [43], MicroMator [44], Navigate [45] and other, Python-based control software [46]). While the extent of demand for APIs among users remains unclear, should this request become more frequent, a Python-based API will be developed using Pybind11 [47], enabling seamless interoperability between the existing C++ software architecture and Python-based workflows.

In conclusion, ClearScope enables high-resolution imaging of intact, cleared specimens at scales previously inaccessible, opening new avenues for investigating neuropsychiatric, neurological and other biological disorders. By overcoming longstanding limitations in whole-organ imaging, the ClearScope system has the potential to accelerate discovery and contribute to the development of novel preventative and therapeutic strategies in neuroscience, oncology and beyond.

## 5. Conclusions

This study describes the development and validation of ClearScope, a fully integrated next-generation light-sheet theta microscopy system that overcomes key technical and practical limitations of existing light-sheet approaches. By combining oblique dual illumination with perpendicular detection, ClearScope enables uniform, subcellular-resolution imaging (with subcellular resolution determined by the lateral performance of the system) of large optically cleared specimens without restricting lateral sample size. The platform integrates optimized hardware, adaptive refractive index mismatch correction and dedicated acquisition and analysis software into a cohesive, user-focused workflow.

Performance evaluations show that ClearScope provides stable image quality across large fields of view, accurate multichannel alignment and sufficient resolution for quantitative applications such as neuron tracing, vascular reconstruction and atlas-based registration. Seamless switching between low- and high-magnification imaging supports efficient multiscale workflows while reducing data volume. Although the system involves trade-offs, including increased illumination demands, the gains in imaging depth, lateral coverage and quantitative fidelity outweigh these limitations. Overall, the ClearScope system offers a practical, scalable solution for high-resolution 3D imaging of intact organs, with broad applicability in neuroscience, developmental biology, cancer research and systems-level tissue studies.

## 6. Patents

The LSTM technology is protected by US Patent US11506877B2 (inventor: Dr. Raju Tomer; assignee: Columbia University, New York, NY, USA) and related, international patent applications (EP3538941A4, WO2018089839A1 and JP2020502558A).

## Figures and Tables

**Table 1:** Full width at half maximum (FWHM) measurements of the ClearScope microscope using an XLPLN10XSVMP objective (10×/0.6 NA ∞, WD = 8 mm; Evident). Details are in the text.

Investigated Region Within the FOV	FWHM [µm]	
	Axial	Lateral
Center	5.81	0.34
Upper-left quarter	6.37	0.36
Lower-left quarter	6.67	0.35
Upper-right quarter	5.45	0.36
Lower-right quarter	5.56	0.37

**Table 2:** Key technical specifications of commercially available light-sheet microscopes and the recently developed, non-commercial Hybrid OTLS (data taken in part from [25]).

System	DetObj	AxRes (µm)	RIR	MD (mm)	MSLS (X Y, mm)
Alpha3 (PhaseView, Seattle, WA, USA) (Figure 15a)	2× (NA = 0.14)	2.0–12.0	1.33–1.56	15	15 × 15 (standard)
	4× (NA = 0.28)				15 × 25 (extended)
	10× (NA = 0.50)				
Blaze (Miltenyi Biotech, Bergisch Gladbach, Germany) (Figure 15a)	1.1× (NA = 0.10)	4.0–24.4	1.33–1.56	17 (1.1×)	24 × 50
	4× (NA = 0.35)			16 (4×)	
	12× (NA = 0.53)			10.9 (12×)	
Lightsheet 7 (Zeiss, Oberkochen, Germany) (Figure 15a)	5× (NA = 0.16)	2.0–14.0	1.33–1.58 (5×)	10	10 × 50
	10× (NA = 0.50)		1.33 (10×)		
	20× (NA = 1.00)		1.33–1.53 (20×)		
	40× (NA = 1.00)		1.33 (40×)		
	63× (NA = 1.00)		1.33 (63×)		
MuVi SPIM CS (Bruker, Billerica, MA, USA) (Figure 15b)	10× (NA = 0.50)	2.0–8.0	1.33–1.51 (10×)	12	12 × 19
	20× (NA = 1.00)		1.44–1.50 (20×)		
AxL Cleared Tissue LightSheet (Intelligent Imaging Innovations) (Figure 15c)	1.0 (NA = 0.25) **	*	1.33–1.56	56 (1.0×)	100 × 30
	1.5 (NA = 0.37) **			30 (1.5×)	
	2.3 (NA = 0.57) **			10 (2.3×)	
SmartSPIM (Lifecanvas, Cambridge, MA, USA) (Figure 15c)	3.6× (NA = 0.20)	1.4–4.0	1.33–1.56	12	20 × 25 (standard)
	15× (NA = 0.40) 22× (NA = 0.70)				30 × 55 (extended)
ct-dSPIM (ASI, Eugene, OR, USA) (Figure 15d)	16× (NA = 0.40)	>1.0	1.33–1.56	5 (16×)	Unconstrained ***
	24× (NA = 0.70)			2 (24×)	
CTLS (Intelligent Imaging Innovations, Denver, CO, USA) (Figure 15d)	1× (NA = 0.20)	3	1.33–1.56	25	25 × 25
	1.5× (NA = 0.37)				
MegaSPIM (Lifecanvas) (Figure 15d)	1.8× *	*	*	*	200 × 200
	3.6× (NA = 0.20)				
	9× *				
	15× (NA = 0.40)				
	22× *				
ClearScope (MBF Bioscience) (Figure 15e)	4× (NA = 0.28)	2.0–6.0	1.33–1.56	17 (4×)	Unconstrained ***
	10× (NA = 0.60)			8 (10×)	
	16× (NA = 0.40)			12 (16×)	
	20× (NA = 1.00)			8 (20×)	
	24× (NA = 0.70)			10 (24×)	
Hybrid OTLS (open-top) [25] (Figure 15f,g)	2× (NA = 0.10)	2.9–15.6	1.33–1.56	10	Unconstrained ***
	24× (NA = 0.70)				

Abbreviations: Ref, reference; DetObj, detection objectives; NA, numerical aperture; AxRes, axial resolution; RIR, refractive index range; MD, maximum depth; MSLS, maximum specimen lateral size; *, information not provided by the manufacturer; ** higher magnification is achieved with a motorized 16:1 zoom in the detection light path by means of a Zeiss Axio Zoom.v16 (Carl Zeiss Microscopy, Oberkochen, Germany); *** lateral size of the specimen unconstrained by the configuration of the illumination and detection objectives. Details are in the text.

## Data Availability

The original contributions presented in this study are included in the article. Further inquiries can be directed to the corresponding author.

## Abbreviations

The following abbreviations are used in this manuscript:

3D	Three-dimensional
LSTM	Light-sheet theta microscopy
LSM	Light-sheet microscopy
SPIM	Selective plane illumination microscopy
CAD	Computer-aided design
ETLs	Electrically tunable lenses
FOV	Field of view
DOC	Detection objective changer
RI	Refractive index
CPU	Central processing unit
GPU	Graphics processing unit
IRIC	Intelligent refractive index compensation
PSF	Point spread function
FWHM	Full width at half maximum
ASLM	Axially swept light-sheet microscopy
API	Application programming interface
